# Alkyltriphenylphosphonium-Functionalized Hyperbranched Polyethyleneimine Nanoparticles for Safe and Efficient Bacterial Eradication: A Structure–Property Relationship Study

**DOI:** 10.3390/ijms26115153

**Published:** 2025-05-28

**Authors:** Katerina N. Panagiotaki, Kyriaki-Marina Lyra, Aggeliki Papavasiliou, Dimitris Tsiourvas, Zili Sideratou

**Affiliations:** Institute of Nanoscience and Nanotechnology, National Centre of Scientific Research ‘‘Demokritos”, 15310 Aghia Paraskevi, Attiki, Greece; k.panagiotaki@inn.demokritos.gr (K.N.P.); k.lyra@inn.demokritos.gr (K.-M.L.); a.papavasiliou@inn.demokritos.gr (A.P.); d.tsiourvas@inn.demokritos.gr (D.T.)

**Keywords:** bactericides, hyperbranched polymers, triphenylphosphonium cation, nanoparticles

## Abstract

Polymeric antibacterial agents are attracting attention due to their increased bactericidal efficiency and low probability of causing drug resistance. Their activity, usually attributed to electrostatic interactions and subsequent disruption of cell membranes, is attributed to the number and chemical structure of their functional groups. In this study, hyperbranched polyethyleneimines (PEIs) of two different molecular weights were functionalized with amphiphilic alkyltriphenylphosphonium groups, which are known to induce membrane penetration, especially in cells with high membrane potential. The obtained nanoparticles were chemically and physicochemically characterized, and their inhibition potential against Gram (−) *E. coli* and Gram (+) *S. aureus* bacteria was determined. The effects of polymer molecular weight, alkyl chain length, and the number of triphenylphosphonium groups on their antimicrobial efficacy were studied. All compounds exhibited antibacterial properties, especially against *S. aureus* (MIC < 50 μg/mL). Low-molecular-weight polymeric derivatives and longer alkyl chains proved more efficient against both *E. coli* (MIC = 20 μg/mL) and *S. aureus* (MIC = 0.25 μg/mL). SEM images depicted changes in cell morphology, bacterial membrane disruption, and leakage of intracellular contents, signifying loss of cell viability. Minimal cytotoxicity against three mammalian cell lines at relevant antibacterial concentrations demonstrated the potential of a structure–property relationship approach for novel potent antibacterial polymers.

## 1. Introduction

Reports from the World Health Organization (WHO) have highlighted the ever-increasing resistance of bacteria over the years [1], purporting antibiotic resistance to be a severe clinical and public health problem [2]. In addition, the WHO is also providing evidence of worldwide overuse of antibiotics during the coronavirus disease (COVID-19) pandemic, which may have also further aggravated antimicrobial resistance [3]. It is, therefore, not surprising that the development of new classes of antibiotics with different/novel modes of action compared to those of already established and widely used antibiotics is currently the main area of research in many laboratories, with each following a variety of approaches towards the final objective, i.e., to successfully challenge resistant pathogens.

Since currently used antibiotics act against well-known molecular targets [4], which have been exploited by bacteria to develop resistance mechanisms, a large part of the current research is considering totally new approaches. The most evident basic requirement of new antibiotics is to have a strong affinity towards the bacterial cell wall and the ability to then penetrate it, or severely disrupt it, while concomitantly being considerably less potent to host cells in order to minimize host cell toxicity. This strong affinity to the bacterial cell wall is typically explored by employing positively charged molecules, macromolecules, peptides, or nanoparticles that are anticipated to strongly interact with the negatively charged components of either Gram-positive or Gram-negative bacterial cell walls. Indeed, it is well known that the surface charge of bacteria is negative due to the presence of carboxylate or phosphate moieties [5,6,7]. The cell walls of Gram-positive bacteria and the outer membranes of Gram-negative bacteria contain anionic lipids and teichoic and lipoteichoic acids, while the plasma membrane also encompasses negatively charged phospholipids and fatty acids [8].

Transversing or penetrating bacterial cells intuitively points to lipophilic compounds, which appears to contradict the need for positively charged entities to initially target bacteria. In this context, it is of interest to note that the efforts to develop new antibiotics that began in the 1990s failed, owing to issues, e.g., in identifying compounds that could effectively penetrate bacterial cell walls, which is accredited to the presence of an effective penetration barrier in bacteria and, in particular, the more restrictive envelope of Gram-negative bacteria [4].

However, over the last few decades, so-called delocalized lipophilic cations (DLCs), a class of low-molecular-weight moieties that are lipophilic and also carry a permanent positive charge, which is delocalized over a number of conjugated double bonds or aromatic rings, have emerged. These lipophilic moieties were found to be able to permeate the hydrophobic membrane barriers easily, taking advantage of their highly negative inner-membrane potential values [9,10,11,12,13]. Due to the high membrane potential, delocalized cations may be moved across lipid bilayers electrophoretically [14,15]. Their main significance and utility are that they can effectively induce the transport of molecules, polymers, peptides, molecular assemblies, or nanoparticles to which they are conjugated through plasma and mitochondrial membranes [9,10,11]. The most prominent among DLCs is the triphenylphosphonium group (TPP), a positively charged chemical moiety that is normally attached at one end of an alkyl chain to confer additional lipophilicity [16,17,18,19]. Its main application is in the field of mitochondrial targeting by taking advantage of the high mitochondrial membrane potential (in the range of 120–180 mV), which results in an increase in the concentration of DLCs in the mitochondrial matrix by up to 100−1000-fold [12,13,14,15,20].

Based on the same principle, TPP has been utilized for the effective penetration of bacterial cells, since, as discussed above, efficient bacterial cell penetration has been considered to be of major importance in the search for novel antibacterials. This notion is based on phylogenetic similarities in the bioenergetics of mitochondria and bacteria according to the theory of the bacterial origin of mitochondria [21,22,23,24,25], which resulted in both having approximately the same elevated negative electrochemical membrane potential (in the order of about −180 mV), which is needed to drive crucial processes in either bacteria or mitochondria [24,25,26,27,28]. Accordingly, over the last decade, TPP conjugation to antibacterial agents/antibiotics or antioxidants, usually through the use of an alkyl chain spacer, has been rather extensively studied, affording promising results. Antibacterial agents such as photosensitizers [29], dihydrocinnamic derivatives [27], chloramphenicol [30,31], or betulin [32] were covalently linked with alkyltriphenylphosphonium moieties of various chain lengths and studied against both Gram (−) and Gram (+) bacteria, and therefore, their action was attributed to their internalization and suppression of cell functions [33,34]. In addition, several groups have studied the effect of the alkyl chain spacer length on the antibacterial efficacy of the TPP conjugates, since, as already mentioned above, not only the delocalized cationic charge but also the lipophilicity of these compounds is important [27,30,34,35]. Overall, the reports suggest that alkyl linkers with chain lengths of about 10 to 16 carbon atoms have a favorable antibacterial effect compared to short-chain alkyl spacers, due to the increase in the lipophilic character. In this respect, it is also of interest to note that even simple alkylTPP derivatives have been efficient against *Escherichia coli* (*E. coli*) bacteria, which was also attributed to their enhanced permeability of bacterial coatings [24].

Given that bacteria have acquired resistance to widely used low-molecular-weight antibiotics, it has been suggested that novel antimicrobial polymers with new modes of antibacterial action, which are also endowed with chemical stability and non-volatility, can provide long-term activity, thus improving antimicrobial efficacy, avoiding bacterial resistance, and reducing residual toxicity [36,37,38]. Therefore, further progress towards novel antibacterial compounds is being pursued by developing oligomeric or polymeric materials with TPP groups that also bear antibacterial modalities. Such materials, obtained either by polymerization of TPP-containing monomers or by covalently attaching TPP moieties to synthetic or natural polymers, were developed to provide prolonged stability, long-term antimicrobial efficacy, and reduced residual toxicity [39,40,41,42,43,44,45,46]. Among the various polymeric architectures, dendritic polymers, including dendrimers and hyperbranched polymers, are advantageous in this respect, since they have a near-spherical structure, a large number of readily available and easily functionalized end groups, and thus have recently been exploited as antibacterial agents [36,47,48,49,50,51]. Among the hyperbranched polymers, polyethyleneimine (PEI) is one of the most promising due to its availability, low cost, and ease of functionalization, while its positive charge, due to the presence of primary, secondary, and tertiary amino groups, makes it ideal for targeting bacteria. For this reason, it was studied as a possible antibacterial agent, and it was established that, due to its polycationic amphiphilic character that enables its interaction with the anionic components of cell envelop, it exhibits antibacterial properties against both Gram (+) and Gram (−) bacteria inducing membrane permeabilization [52,53]. PEI functionalization with quaternary groups has also been exploited and shown antibacterial efficiency [54,55,56,57], while functionalization with both quaternary groups and hydrophobic alkyl or aromatic groups endows a cationic amphiphilic character to the polymers, resulting in a more effective interaction with the cell envelope, further enhancing its antibacterial activity [58,59]. Quaternized alkylated PEI-based nanoparticles were also synthesized and incorporated into dental composite resins, which showed strong antibacterial activity without leaching out for prolonged periods of time [60,61]. Furthermore, PEI functionalization with guanidinium groups has yielded hyperbranched polymers that have antibacterial properties and also induce excellent dispersibility of carbon nanodisks in water, affording hybrid materials of enhanced antibacterial activity against Gram (−) *E. coli* and Gram (+) *S. aureus* bacteria [62].

**Scheme 1:**
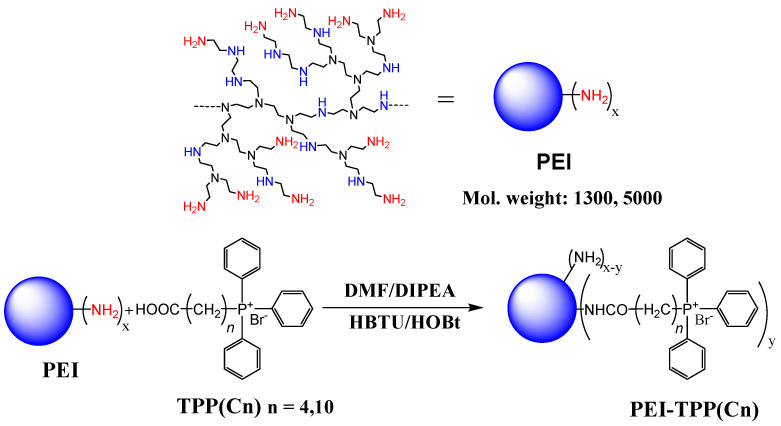
PEI functionalization reactions for the synthesis of PEI-TPP(C4) and PEI-TPP(C10) derivatives.

Our group previously developed TPP-functionalized drug delivery systems based on dendritic polymers or carbon nanodots capable of targeting mitochondria and thus was able to demonstrate the efficient delivery of drugs into the mitochondria of cultured cells or cancer stem-like cells cultured in 3D cell cultures [63,64,65]. In this study, the primary amino groups of hyperbranched polyethyleneimines (PEIs) of two different molecular weights (1300 and 5000 Da) were functionalized with alkyl-triphenylphosphonium (alkylTPP) functional groups of two different alkyl chain lengths (C4, C10), as shown in Scheme 1. Following their physicochemical characterization, we evaluated their antibacterial properties against Gram (−) *E. coli* and Gram (+) *S. aureus* bacteria, by monitoring bacterial growth inhibition, and determining minimum inhibitory concentration (MIC) and minimum bactericidal concentration (MBC), employing broth macro-dilution and colony-counting methods, respectively. Scanning electron microscopy (SEM) studies were used to evaluate the morphology of bacteria after treatment with alkylTPP-functionalized PEI (PEI-TPP) derivatives. In vitro cytotoxicity studies against mammalian cell lines were also performed to investigate their safety at MIC-related concentrations. By synthesizing a number of alkylTPP derivatives of PEI, this structure–property relationship study was the first attempt to determine the route to optimizing antibacterial properties with concurrent non-toxicity against mammalian cells.

## 2. Results and Discussion

### 2.1. Synthesis and Characterization of PEI-TPP Derivatives

The chemical structure of the parent hyperbranched polyethyleneimines of two different molecular weights (1300 and 5000 Da), referred to as PEI1300 and PEI5000, respectively, was initially characterized employing inverse-gated decoupling ^13^C NMR spectroscopy, and their primary-to-secondary-to-tertiary amine ratio and degree of branching were determined as previously described in the literature [66]. It was found that the primary-to-secondary-to-tertiary amine ratios of PEI1300 and PEI5000 were 1.00:1.08:0.74 and 1.00:1.10:0.96, respectively, while the degree of branching was 0.67 for both polymers. The average number of primary amino groups is 10 and 38 per PEI1300 and PEI5000 scaffold, respectively.

The introduction of butyl- or decyl- triphenylphosphonium groups (TPP(C4) or TPP(C10), respectively) onto the PEI1300 and PEI5000 scaffolds was achieved via a coupling reaction between the primary amines of PEI and either (4-carboxybutyl)triphenylphosphonium bromide or (10-carboxydecyl)triphenylphosphonium bromide in an alkaline environment, following a protocol similar to that described in our previous publications (Scheme 1) [63,64,65,67]. By carefully selecting the molar ratio of the reactants and controlling the synthetic conditions, it is feasible to adjust the number of akylTPP groups that are linked to each PEI scaffold. The chemical structures of the final products, i.e., PEI1300-TPP(C4), PEI5000-TPP(C4), PEI1300-TPP(C10), and PEI5000-TPP(C10), were established by proton and carbon NMR (Figures S1–S4) as well as FTIR (Figure S5) spectroscopies.

In detail, two new peaks at ~3 ppm and 2.10 ppm were observed in the ^1^H NMR spectra of all derivatives (Figures S1–S4), attributed to the protons of methylene groups relative to the newly formed amide groups, confirming the successful coupling between the carboxyl groups of (4-carboxybutyl)triphenylphosphonium bromide or (10-carboxydecyl)triphenylphosphonium bromide and the primary amino groups of PEI. Additionally, peaks corresponding to the aromatic protons of the TPP group (7.60–7.90 ppm), the methylene protons adjacent to P^+^ (3.40 ppm), and alkyl chain protons (peaks in the region between 1.10 and 1.70 ppm) were also observed, confirming the presence of alkylTPP groups in the final products. Additionally, the successful synthesis of PEI-TPP derivatives was validated by ^13^C NMR spectroscopy (Figures S1–S4). Specifically, the successful introduction of the alkylTPP groups to the PEI scaffold was confirmed by the peaks at (a) 175 ppm, attributed to the carbons of newly formed amide moieties, and (b) ~36 ppm, attributed to the α-methylene carbons of the alkyl chains relative to the amide groups. Finally, to determine the average number of alkylTPP groups per PEI scaffold, the integration of the ^1^H NMR peak attributed to the aromatic protons of TPP (7.60–7.90 ppm) was compared to that of the peak attributed to the PEI backbone protons (2.45–2.90 ppm). The analysis revealed that, on average, 5.8_±0.5_ and 28.7_±0.4_ butylTPP groups were attached to PEI1300 and PEI5000, respectively, while 2.8_±0.3_ and 34.2_±0.5_ decylTPP groups were attached to PEI1300 and PEI5000, respectively. These functionalization degrees were found to be optimal for achieving a suitable hydrophilic/lipophilic balance, rendering the resulting derivatives insoluble in water yet capable of forming nanoparticles in aqueous media, such as PBS or Opti-MEM (see below in Section 2.2).

Furthermore, FTIR spectroscopy was used to further support the successful introduction of alkylTPP groups to PEI. Specifically, the successful formation of the amide groups was confirmed by the characteristic bands at 1650 cm^−1^ (Amide I), 1545 cm^−1^ (Amide II) and 1240 cm^−1^ (Amide III) [68]. Additionally, the introduction of alkylTPP groups was established by the characteristic bands of alkyl chains at 2940 and 2850 cm^−1^ attributed to the asymmetric and symmetric vibrations of CH_2_, respectively; bands at 1435 and 725 cm^−1^ were assigned to CH_2_ bending and rocking vibrations, respectively; the characteristic bands of TPP aromatic groups at 1455 and 750 cm^−1^ were ascribed to C=C stretching and CH wagging vibrations, respectively; and the band at 690 cm^−1^ was assigned to C–C–C ring-bending vibrations. Also, the bands at 835, 511, and 495 were attributed to P^+^-C stretching vibrations [65,69].

### 2.2. Preparation and Characterization of PEI-TPP Nanoparticles

The as-prepared PEI-TPP derivatives are insoluble in water; however, they are able to form stable colloidal dispersions in various media, such as PBS buffer or Opti-MEM. Thus, PEI-TPP nanoparticles were prepared by adding 100 μL of a methanolic PEI-TPP solution to 900 μL Opti-MEM under vigorous stirring. PEI-TPP nanoparticles were spontaneously obtained and characterized by dynamic light scattering (DLS) and *ζ*-potential measurements to determine their size and charge, respectively. The results are summarized in Table 1. As observed in Figure S6 and Table 1, both nanoparticles of the lower-molecular-weight PEI-TPP had smaller mean radii (40–80 nm) than those of higher molecular weight (~150 nm), regardless of the alkyl chain length. This indicates that the polymer molecular weight is the critical factor affecting nanoparticle self-assembly. The *ζ*-potential values followed the same trend, i.e., PEI1300-TPP(C4) and PEI1300-TPP(C10) nanoparticles exhibited lower positive ζ-potential values (~30–37 mV) than those of PEI5000-TPP(C4) and PEI5000-TPP(C10) nanoparticles, which reached ~60 mV, likely due to the higher TPP content on the nanoparticle surface.

### 2.3. Antibacterial Properties of alkylTPP-Functionalized PEI Nanoparticles

**Figure 1:**
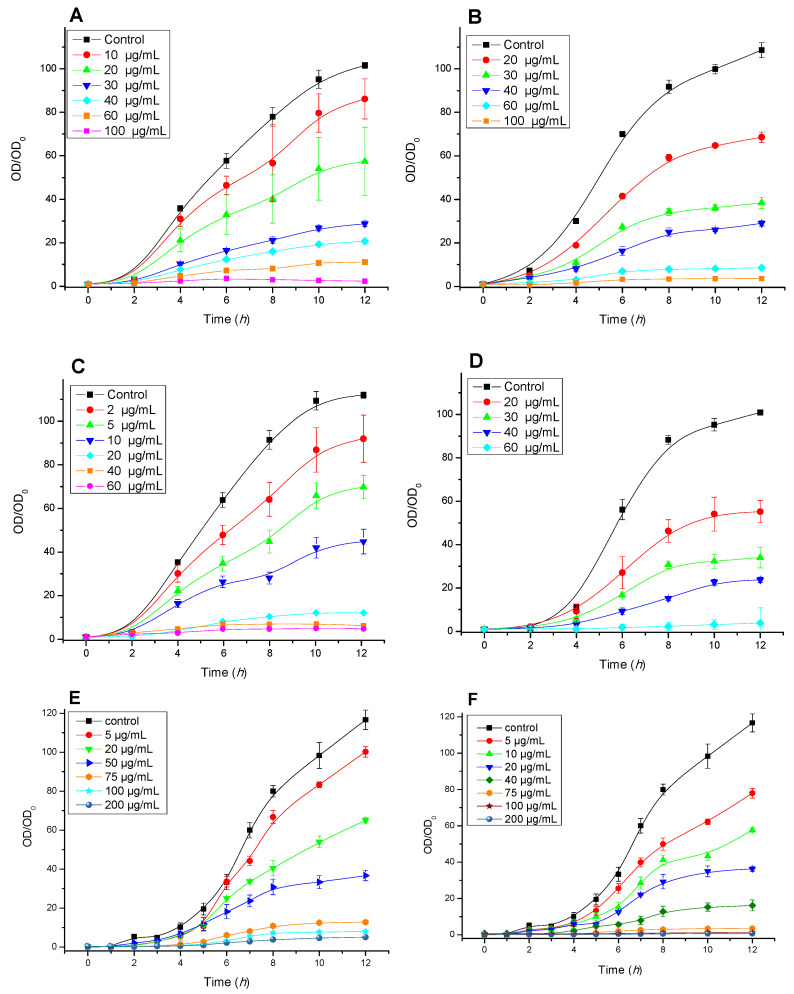
Growth curves of *Escherichia coli* bacteria in the absence (control) or presence of PEI1300-TPP(C4) (**A**), PEI5000-TPP(C4) (**B**), PEI1300-TPP(C10) (**C**) and PEI5000-TPP(C10) (**D**) as well as of the parent polymers, PEI1300 (**E**) and PEI5000 (**F**).

**Figure 2:**
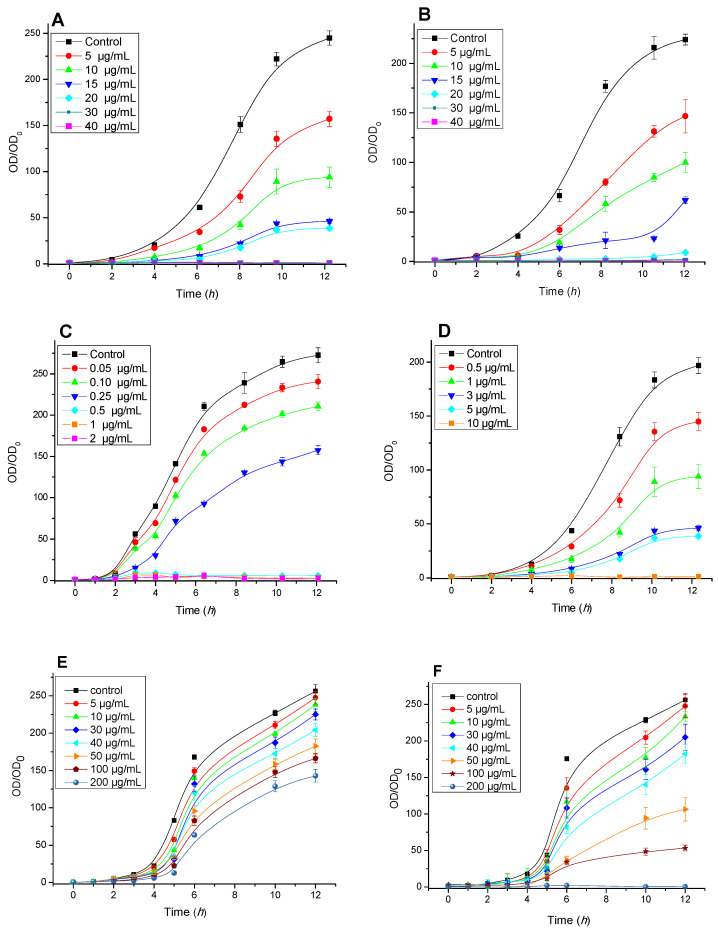
Growth curves of *Staphylococcus aureus* bacteria in the absence (control) or presence of PEI1300-TPP(C4) (**A**), PEI5000-TPP(C4) (**B**), PEI1300-TPP(C10) (**C**) and PEI5000-TPP(C10) (**D**) as well as of the parent polymers, PEI1300 (**E**) and PEI5000 (**F**).

The antibacterial efficacy of alkylTPP-functionalized PEI nanoparticles was assessed against two bacterial strains, i.e., Gram (+) *Staphylococcus aureus* (*S. aureus*) and Gram (−) *Escherichia coli* (*E. coli*) bacteria. For comparison, the parent PEI derivatives were also evaluated under the same conditions. Initially, the study focused on monitoring the bacterial growth rate in the presence of PEI-TPP nanoparticles at concentrations ranging from 0.05 μg/mL to 100 μg/mL, or of parent PEI derivatives at concentrations ranging from 5 μg/mL to 200 μg/mL, by measuring the optical density (OD) of the treated cultures over a 12-h period. Figure 1 and Figure 2 depict the growth kinetics curves of *E. coli* and *S. aureus* bacteria, respectively, as a function of OD/OD_0_ ratio vs. incubation time. As shown, the logarithmic growth rate of both strains decreased in a dose-dependent manner, suggesting that both PEI derivatives as well as all PEI-TPP nanoparticles prevent bacterial growth, albeit at different rates. In more detail, the growth rate of *E. coli* bacteria treated with PEI1300-TPP(C4) or PEI5000-TPP(C4) was significantly reduced at concentrations between 20 and 40 μg/mL compared to the control, while higher concentrations (60 or 100 μg/mL) resulted in near-complete inhibition of bacterial growth (Figure 1A,B). Similar inhibition rates of bacterial growth after treatment with PEI5000-TPP(C10) were observed, as a considerable reduction in the bacterial growth rate was detected in the concentration range between 20 and 40 µg/mL, while complete growth inhibition was registered at concentrations above 60 µg/mL (Figure 1D). In contrast, PEI1300-TPP(C10) exhibited greater activity against *E. coli* bacteria compared to the other derivatives, with almost complete growth inhibition at just 20 μg/mL (Figure 1C). On the other hand, *E. coli* cultures treated with the parent PEI1300 and PEI5000 showed moderate growth inhibition at concentrations between 5 and 50 μg/mL (Figure 1E,F); however, complete bacterial growth inhibition was only observed at considerably higher concentrations, i.e., at 200 and 100 μg/mL for PEI1300 and PEI5000, respectively. Thus, PEI1300-TPP(C4) (Figure 1A) and PEI1300-TPP(C10) (Figure 1C) demonstrated faster and more effective inhibition of *E. coli* growth than the parent PEI1300, while PEI5000-TPP(C10) (Figure 1D) exhibited slightly better performance than both PEI5000-TPP(C4) and PEI5000 (Figure 1B,F).

On the other hand, all PEI-TPP derivatives exhibited increased activity against *S. aureus* compared to *E. coli* bacteria (Figure 2). Specifically, as shown in Figure 2A,B, the growth rates of *S. aureus* bacteria treated with PEI1300-TPP(C4) or PEI1300-TPP(C4) at concentrations ranging from 5 to 20 μg/mL or 5 to 20 μg/mL, respectively, were significantly reduced compared to the control, while complete bacterial growth inhibition was observed at slightly higher concentrations (30–40 μg/mL or 20–40 μg/mL, respectively). Furthermore, bacterial growth was suppressed by PEI5000-TPP(C10) at concentrations as low as 0.5 to 5 μg/mL (Figure 2D), with complete inhibition registered at 10 μg/mL. Interestingly, a reduction in bacterial growth was observed when *S. aureus* bacteria were exposed to very low concentrations of PEI1300-TPP(C10), i.e., 0.05–0.25 μg/mL, and no noticeable bacterial growth was detected following treatment with just 1 μg/mL for 12 h (Figure 2C). In comparison with the parent PEI polymers, all PEI-TPP derivatives significantly reduced bacterial growth kinetics. As shown in Figure 2E,F, complete growth inhibition was achieved with 200 μg/mL of PEI5000, whereas only 50% growth inhibition was observed with the same concentration of PEI1300.

Overall, it can be concluded that, among all tested PEI-TPP derivatives and parent polymers, PEI1300-TPP(C10) exhibited the most significant antibacterial properties against both tested microorganisms, as the lowest concentration of PEI1300-TPP(C10) was required to completely inhibit bacterial cell growth.

In addition to the above, the minimum inhibitory concentration (MIC) and minimum bactericidal concentration (MBC) values of parent PEI polymers and PEI-TPP derivatives against *E. coli* and *S. aureus* bacteria were determined using the broth macro-dilution and colony-counting methods, following the M07-A9 and M26-A protocols, respectively, published by the Clinical Laboratory Standards Institute (CLSI) [70,71]. As shown in Table 2, the MIC values for parent PEI1300 and PEI 5000 against *E. coli* were 150 and 300 μg/mL, respectively, with corresponding MBC values of 400–500 μg/mL. Against *S. aureus*, the parent polymers were slightly more effective, with MIC = 100 μg/mL and MBC = 300 μg/mL. On the other hand, the MIC values of PEI1300-TPP(C4) and PEI5000-TPP(C4) were 150 μg/mL and 100 μg/mL for *E. coli*, respectively, along with 50 μg/mL and 25 μg/mL, respectively, for *S. aureus*. Additionally, the MBC values of these derivatives were 400 μg/mL and 300 μg/mL for *E. coli*, respectively, as well as 300 μg/mL and 150 μg/mL for *S. aureus*, respectively. These findings suggest that PEI5000-TPP(C4) is more active than PEI1300-TPP(C4) against both tested strains, probably due to its higher positive charge, which facilitates stronger electrostatic interactions with the bacterial cell envelope. On the other hand, in the case of *E. coli* bacteria, the MIC values of PEI1300-TPP(C10) and PEI5000-TPP(C10) were found to be 20 μg/mL and 40 μg/mL, respectively, while the MBC values were 25 μg/mL and 50 μg/mL, respectively. Furthermore, against *S. aureus* bacteria, both derivatives exhibited MIC and MBC values of 0.25 μg/mL and 5 μg/mL, respectively.

Based on the abovementioned results, derivatives bearing decyltriphenylphosphonium groups are more active against both tested microorganisms compared to those bearing butyltriphenylphosphonium moieties or the non-functionalized parent polymers, with PEI1300-TPP(C10) being the most active among them. The antibacterial activity of these derivatives is thus closely related to the presence of TPP cations, which facilitate electrostatic and hydrophobic interactions with negatively charged components of the bacterial cell envelope, such as phospholipids or peptidoglycan, and easily penetrate biological protein–lipid membranes, affecting their structural integrity and functionality. It is also related to the length of alkyl linkers, which plays a critical role in defining the hydrophobic/hydrophilic balance of polymeric derivatives. These findings are in accordance with previous studies reporting that the presence of linkers with long-alkyl chain lengths, ranging from 10 to 16 carbon atoms between the amide group and the TPP cation, induces enhanced antibacterial activities in contrast to short-chain linkers [27,34,72]. The hydrophilic/hydrophobic balance of TPP-functionalized nanoparticles has been identified as a key factor, with numerous studies in the literature correlating their accumulation, toxicity, or drug efficacies with lipophilicity [15,18,24,73,74]. Although the size and *ζ*-potential of TPP-functionalized nanoparticles also contribute, their effect appears less significant. For instance, both PEI5000-TPP nanoparticles have the same mean radii and *ζ*-potential values, but significantly different toxicities especially against *S. aureus*. Similarly, among the PEI1300-TPP nanoparticles, the C10 derivative is the most potent, although it has the lowest (less positive) ζ-potential value. Another point that should be noted is that Gram (+) bacteria seem to be more sensitive to these PEI-TPP derivatives, particularly to the PEI1300-TPP(C10) and PEI5000-TPP(C10) derivatives, compared to Gram (−) bacteria. This increased sensitivity could be attributed to the difference between the cell wall structures of Gram (+) and Gram (−) bacteria, as well as the membrane compositions and the barrier of the two membranes of Gram (−) bacteria compared to the single membrane of Gram (+) bacteria. Similar findings have been reported in other works, where alkylTPP-functionalized compounds were found to be more active against Gram (+) than Gram (−) bacteria [32,34,72,75].

**Figure 3:**
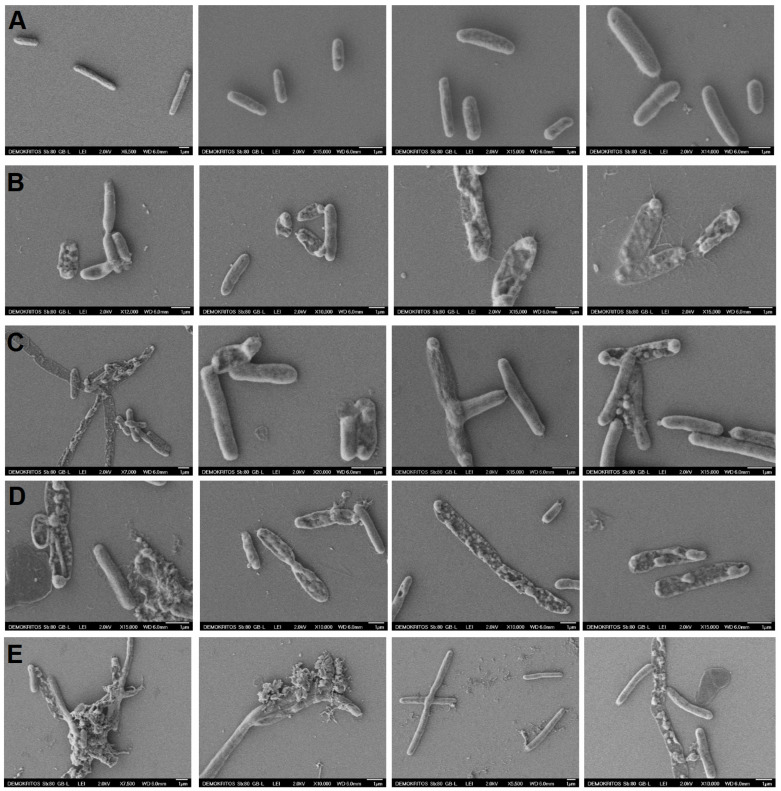
SEM images of *E. coli* bacteria: untreated cells (**A**) and cells after 4 h incubation time at 37 °C with PEI1300-TPP(C4) (**B**), PEI5000-TPP(C4) (**C**), PEI1300-TPP(C10) (**D**), and PEI5000-TPP(C10) (**E**) at ½ MIC. In all micrographs, the scale bar is 1 μm.

**Figure 4:**
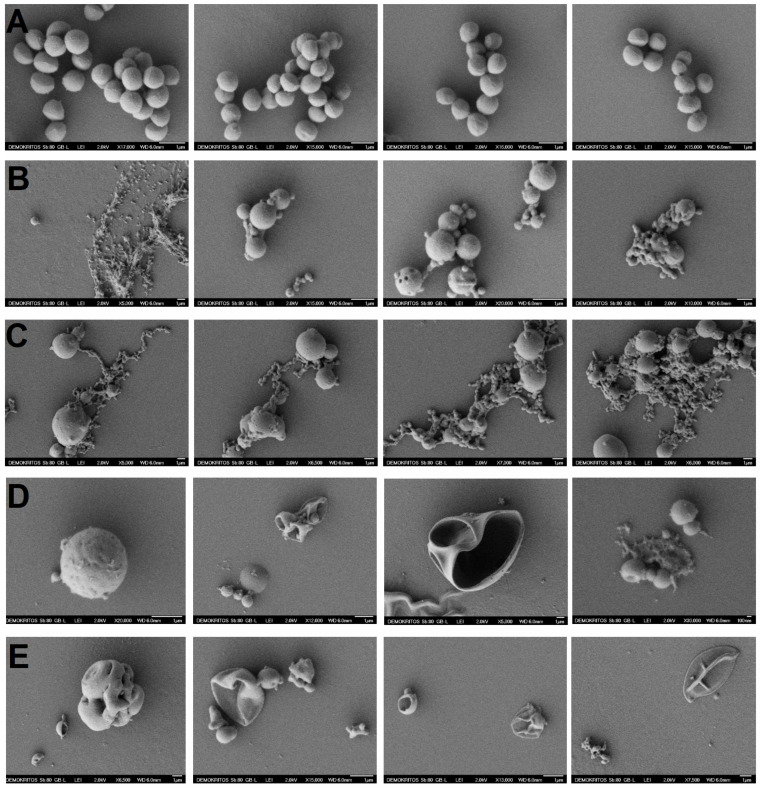
SEM images of *S. aureus* bacteria: untreated cells (**A**) and cells after 4 h incubation time at 37 °C with PEI1300-TPP(C4) (**B**), PEI5000-TPP(C4) (**C**), PEI1300-TPP(C10) (**D**), and PEI5000-TPP(C10) (**E**) at ½ MIC. In all micrographs, the scale bar is 1 μm.

Furthermore, the morphology of *E. coli* and *S. aureus* bacteria after treatment with PEI-TPP derivatives for 4 h at 37 °C was investigated using scanning electron microscopy (SEM). Both untreated bacteria (control) and bacteria treated with PEI-TPP derivatives at ½ MIC are shown in Figure 3 and Figure 4. The untreated bacteria appear healthy, having a smooth and intact surface, and as expected, the *E. coli* and *S. aureus* bacteria have typical rod and spherical shapes, respectively (Figure 3A and Figure 4A). In contrast, the effect of PEI-TPP derivatives on *E. coli* and *S. aureus* bacteria was found to result in significant changes in cell morphology, as observed in Figure 3B–E and Figure 4B–E, respectively. Specifically, the treatment of both tested bacteria with the PEI-TPP derivatives revealed a loss of membrane integrity, as cells shrank and their surface appeared rough with furrows and blebs, while in many cases, cell walls and cytoplasmic membranes were ruptured. Deformation of cell shape was also evident as cells appeared irregular, flattened, or wrinkled, while some images clearly showed the leakage of intracellular contents, indicating a loss of cell viability. Notably, some *E. coli* bacteria appeared to be elongated and some *S. aureus* bacteria appeared inflated or deflated compared to the untreated cells, which is a typical response of bacteria to stress. Similar results have been reported in the literature, where alkylTPP-functionalized compounds caused rupture of the bacterial cell wall and/or membrane, followed by a pronounced release of cytoplasmic components and thus a complete loss of cell viability [76,77,78]. The exact mechanism of the antibacterial activity of alkylTPP-functionalized compounds has not been fully interpreted [72], but it has been reported to be related to their ability to accumulate on the cell membrane or form a monolayer around the cell due to strong electrostatic and hydrophobic interactions. These interactions may alter the membrane potential, causing its destabilization and inducing local membrane rupture with leakage of intracellular contents and/or pore formation [27]. Following this step, alkylTPP-functionalized compounds are easily internalized into bacterial cells, causing alterations in bacterial metabolism [28,33]. Herein, the action mechanism of PEI-TPP derivatives may involve a sequence of events, starting with their interaction with the cell envelope, followed by destabilization of membrane integrity, efficient internalization into bacterial cells, alteration of bacterial metabolic activity, and ultimately cell death.

### 2.4. In Vitro Cytotoxic Evaluation of alkylTPP-Functionalized PEI Nanoparticles

**Figure 5:**
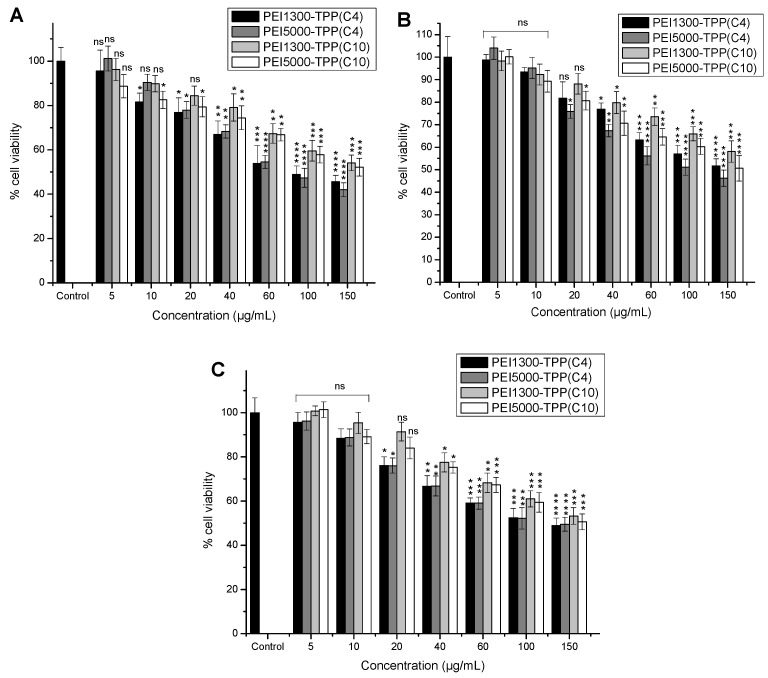
Comparative toxicities of PEI-TPP derivatives on HEK293 (**A**), PC3 (**B**), and DU145 (**C**) cells following incubation at various concentrations for 3 h as determined by the MTT assay following 24 h incubation. Data are expressed as the mean ± SD of six independent values obtained from at least three independent experiments. The statistical significance, obtained from Student’s paired two-tailed *t*-tests, follows the assignment * *p* < 0.05, ** *p* < 0.01, *** *p* < 0.001, **** *p* < 0.0001, and ns, not significant.

As toxicity is a critical factor in selecting a suitable and effective antibacterial agent in various fields, the cytotoxicity of the alkylTPP-functionalized PEI nanoparticles was assessed against human embryonic kidney HEK293 cells as well as human prostate PC3 and DU145 cells. Thus, cells were incubated with PEI-TPP derivatives at concentrations ranging from 5 to 150 μg/mL for 3 h, and cell viability was assessed at 24 h post -incubation using the well-known MTT assay. As shown in Figure 5, a dose-dependent cytotoxic effect on the tested cell lines was observed for all PEI-TPP derivatives. At concentrations up to 20 μg/mL, all derivatives exhibited minimal cytotoxicity (cell viability > 80%) even after a 24 h incubation time. Derivatives bearing butylTPP groups were found to be slightly more toxic (IC_50_ values of 60–100 μg/mL) than the derivatives with decylTPP groups (IC_50_ ≥ 150 μg/mL). It should be noted that the polymeric derivative exhibiting the highest antibacterial activity, i.e., PEI1300-TPP(C10), can be considered safe at relevant antibacterial concentrations (MIC = 20 μg/mL and 0.25 μg/mL for *E. coli* and *S. aureus*, respectively) as the corresponding cell viability values after a 24 h incubation time are not statistically significant compared to the controls.

## 3. Materials and Methods

### 3.1. Chemicals and Reagents

Hyperbranched polyethyleneimines (PEIs) of two different molecular weights, i.e., 1300 Da (Lupasol^®^ G20, water-free, 98%) and 5000 Da (Lupasol^®^ G100, water-free, 99%), were kindly donated by BASF (Ludwigshafen, Germany). *N*,*N*-diisopropylethylamine (DIPEA), (4-carboxybutyl)triphenylphosphonium bromide, thiazolyl blue tetrazolium bromide (MTT), sodium chloride (NaCl), tryptic soy broth (TSB), glutaraldehyde (solution, 25%), and sodium cacodylate were purchased from Sigma-Aldrich Ltd. (Poole, UK). *N*-hydroxybenzotriazole (HOBt) and 2-(1*H*-benzotriazole-1-yl)-1,1,3,3-tetramethyluronium (HBTU) were purchased from Anaspec (San Jose, CA, USA). RPMI 1640 medium with phenol red, penicillin/streptomycin, fetal bovine serum (FBS), phosphate-buffered saline (PBS), L-glutamine, and trypsin/EDTA were purchased from Biowest (Nuaillé, France). Opti-MEM without phenol red (Gibco, Grand Island, NY, USA) was obtained from ThermoFisher Scientific (San Jose, CA, USA). Peptone from casein was purchased from AppliChem GmbH (Darmstadt, Germany), while agar and Luria–Bertani broth (LB) were obtained from MP Biomedicals (Illkirch, France). High-purity solvents such as *N*,*N*-dimethylformamide (DMF, anhydrous, 99.8%), ethanol (99.9%), methanol (≥99.8%), and isopropanol (99.8%) were obtained from Merck KGaA (Calbiochem^®^, Darmstadt, Germany).

### 3.2. Synthesis of PEI-TPPs

The introduction of the butyl-triphenylphosphonium or decyl-triphenylphosphonium groups to PEI of two different molecular weights, i.e., 1300 Da and 5000 Da, was achieved following a similar synthetic procedure to that described in our recent publication [63,64,65,67]. Specifically, to an anhydrous DMF solution (10 mL) containing DIPEA (4 mmol) and either the commercially available (4-carboxybutyl)triphenylphosphonium bromide (1 mmol) or (10-carboxydecyl)triphenylphosphonium bromide (1 mmol), synthesized by the reaction of triphenylphosphine with 11-bromoundecanoic acid in dry DMF as described in our previous publication [67], an anhydrous DMF solution (3 mL) containing HOBt (1.2 mmol) and HBTU (1.2 mmol) was added and allowed to react at room temperature for 1 h under an inert atmosphere (Scheme 1). Subsequently, PEI (0.4 mmol) dissolved in anhydrous DMF (4 mL) was added, and the reaction mixture was allowed to react overnight at room temperature under an inert atmosphere. The solvent was partially removed under a vacuum, and the crude product was obtained after precipitation with diethyl ether (twice) and Opti-MEM media (twice). The final products, i.e., PEI1300-TPP(C4), PEI5000-TPP(C4), PEI1300-TPP(C10) and PEI5000-TPP(C10), were obtained after drying under vacuum, and their structures were verified by ^1^H and ^13^C NMR (Bruker Avance DRX spectrometer, Bruker Biospin, Rheinstetten, Germany, operating at 500 and 125.1 MHz, respectively) and FTIR spectroscopy (Nicolet 6700 spectrometer, Thermo Scientific, Waltham, MA, USA, coupled with a Specac Quest ATR with a diamond crystal, Specac Ltd., London, UK, at 4 cm^−1^ resolution).

^1^H NMR (500 MHz, MeOD-*d*_4_):

For PEI1300-TPP(C4), PEI5000-TPP(C4): δ = 1.60 (t, CH_2_CH_2_P^+^Ph_3_), 1.80 (t, NHCOCH_2_CH_2_), 2.10–2.20 (t, NHCOCH_2_), 2.45–2.90 (m, CH_2_ of PEI skeleton), 3.10–3.20 (CH_2_NHCO), 3.3–3.40 (t, CH_2_P^+^Ph_3_), 7.60–7.90 (m, aromatic H).

For PEI1300-TPP(C10), PEI5000-TPP(C10): δ = 1.10–1.30 (m, aliphatic CH_2_), 1.40–1.70 (m, CH_2_CH_2_P^+^Ph_3_, NHCOCH_2_CH_2_), 2.10–2.20 (t, NHCOCH_2_), 2.45–2.85 (m, CH_2_ of PEI skeleton), 2.90–3.05 (CH_2_NHCO), 3.40–3.45 (m, CH_2_P^+^Ph_3_), 7.60–7.80 (m, aromatic H).

^13^C NMR (125.1 MHz, MeOD-*d*_4_):

For PEI1300-TPP(C4), PEI5000-TPP(C4): δ = 174.8 (C=O), 134.9 (P^+^Ph_3_ para), 133.0 (d, *J =* 9.3 Hz, P^+^Ph_3_ ortho/meta), 130.5 (d, *J* = 12.33 Hz, P^+^Ph_3_ ortho/meta), 120.2 (d, *J* = 10.80 Hz, P^+^Ph_3_ ipso), 54–45 (NCH_2_CH_2_N), 37.8–39.5 (NH_2_CH_2_, CH_2_NHCO), 34.9–35.6 (CONHCH_2_), 24.6–25.1 (CONHCH_2_CH_2_), 22.2 (CH_2_P^+^Ph_3_), 21.5 (CH_2_CH_2_P^+^Ph_3_).

For PEI1300-TPP(C10), PEI5000-TPP(C10): δ = 175.1 (C=O), 135.4 (P^+^Ph_3_ para), 133.4 (d, *J =* 10.3 Hz, P^+^Ph_3_ ortho/meta), 130.2 (d, *J* = 13.47 Hz, P^+^Ph_3_ ortho/meta), 123.0 (d, *J* = 11.67 Hz, P^+^Ph_3_ ipso), 55–49 (NCH_2_CH_2_N), 36.7–40.6 (NH_2_CH_2_, CH_2_NHCO), 35.8 (CONHCH_2_), 30.4 (d, *J* = 17.17 Hz, CH_2_CH_2_CH_2_P^+^Ph_3_), 29.5–28.0 (aliphatic CH_2_), 22.1 (CH_2_P^+^Ph_3_), 21.4 (d, *J* = 31.50 Hz, CH_2_CH_2_P^+^Ph_3_).

FTIR (cm^−1^): 3340 (m) *ν*_as_(NH), 3250 (m) *ν*_s_(NH_2_), 2940 (s) *ν*_s_(CH_2_), 2850 (s) *ν*_as_(CH_2_), 1650 (s) (Amide I), 1545 (m) (Amide II), 1455 (s) *ν*(CC), 1435 (s) *δ*(CH_2_), 1240 (m) (Amide III), 1100(s) *ν*_as_(C-N), 835 (w) *ν*(P^+^-C), 750 (m) γ(CH, aromatic) 725(w) *ρ*(CH_2_), 690 (s) (φ CC), 511 (s) and 495 (s) *ν*_s_(P^+^-C (X-sensitive modes).

### 3.3. Development of PEI-TPP Nanoparticles

PEI-TPP nanoparticles were prepared using a procedure analogous to that described in our previous publications [63,64,67]. In brief, 100 μL of a methanolic PEI-TPP solution (30 mg/mL) was added drop-wise to 900 μL Opti-MEM under vigorous stirring to ensure thorough mixing. Nanoparticles formed spontaneously and were subsequently subjected to further characterization. The size distribution of PEI-TPP nanoparticles was measured using dynamic light scattering (DLS) employing an AXIOS-150/EX apparatus (Triton Hellas, Thessaloniki, Greece) equipped with a 30 mW laser source and an Avalanche photodiode detector at an angle of 90°. For these measurements, 200 μL of each PEI-TPP nanoparticle dispersion was utilized. A minimum of ten light scattering measurements were obtained for each dispersion, and the results were averaged. Autocorrelation functions were collected for 20 s and analyzed using the CONTIN algorithm to obtain the apparent hydrodynamic radius distributions. Furthermore, *ζ*-potential measurements of PEI-TPP nanoparticle dispersions were performed employing a ZetaPlus apparatus (Brookhaven Instruments Corp, Long Island, NY, USA). For these experiments, 200 μL of each PEI-TPP nanoparticle dispersion was diluted with 1.4 mL water, ten *ζ*-potential measurements were collected for each dispersion, and the results were averaged.

### 3.4. Evaluation of PEI-TPP Antibacterial Activities

The antibacterial activity of PEI-TPP nanoparticles was initially evaluated using a bacterial growth inhibition assay and subsequently by the determination of their MIC and MBC values. MIC values were determined using the macro-dilution method, while MBC values were assessed employing the colony-counting method according to the M07-A9 and M26-A protocols published by the Clinical Laboratory Standards Institute (CLSI), respectively [70,71]. Specifically, for these studies, *Escherichia coli* (strain DH5a) and *Staphylococcus aureus* (strain ATCC 25923) bacteria were used as model Gram (−) and Gram (+) bacteria strains, respectively. *E. coli* and *S. aureus* bacteria were cultured in Luria–Bertani (LB) and tryptic soy broth (TSB) medium, respectively, under aerobic conditions, with constant shaking using a Stuart SI500 orbital shaker (~200 rpm shaking speed) at 37 °C for 16 h. Then, the bacterial suspensions were diluted with LB or TSB to a concentration equivalent to 0.5 McFarland Standard, i.e., ~10^8^ colony-forming units per milliliter (CFU/mL), and the obtained bacterial suspensions were utilized for the subsequent tests.

*Bacterial growth study*: *E. coli* and *S. aureus* bacteria were added to PEI-TPP dispersions or to broth media (control) at a final concentration of 10^5^ CFU/mL and PEI-TPP concentrations ranging from 2 to 100 μg/mL, and incubated under constant shaking in a Stuart SI500 orbital shaker (~200 rpm shaking speed, Bibby Scientific Ltd., Staffordshire, UK) for 12 h at 37 °C. Throughout the incubation time, bacterial growth was monitored by recording the optical density (OD) of the bacterial suspensions at a wavelength of 600 nm, every two hours for *E. coli* and every hour for *S. aureus*. Bacterial growth curves were plotted as OD/OD_0_ values versus incubation time.

*MIC and MBC determination*: *E. coli* and *S. aureus* overnight cultures (10^5^ CFU/mL) were inoculated into 10 mL tubes, and subsequently, PEI-TPP dispersions were added at concentrations ranging from 0.25 to 500 μg/mL. Untreated bacteria were used as controls. MIC values were determined as the minimum concentration of polymeric derivatives at which no visible growth of the microorganism was detected after a 24 h incubation period at 37 °C. For MBC determination, a volume of 50 μL of treated bacteria from the MIC experiment was collected from the tubes at the MIC value, as well as from three tubes above the MIC value, and placed onto LB agar plates for 18 h incubation at 37 °C. Subsequently, the number of colonies on the plates was counted, and the MBC endpoint was defined as the minimum concentration at which 99.9% of the initial bacterial inoculum was killed.

*Morphological analysis by scanning electron microscopy*: The morphology of the bacteria after treatment with PEI-TPP nanoparticles was studied using a scanning electron microscope (Jeol JSM 7401F Field Emission SEM, JEOL Ltd., Tokyo, Japan). Briefly, *E. coli* and *S. aureus* bacteria were treated with PEI-TPP nanoparticles at a concentration equal to ½ MIC. After 4 h incubation, bacteria were fixed in a solution of glutaraldehyde (3% in 100 mM sodium cacodylate buffer, pH = 7.1) for 12 h. Afterwards, bacteria were collected, thoroughly washed, and redispersed in the same sodium cacodylate buffer. Then, 50 μL of the fixed bacteria was deposited onto a poly(l-lysine)-coated glass cover slip, and the bacteria were dehydrated using a series of ethanol concentrations (50%, 70%, 95%, and 100%) for 10 min each. After drying, the sample was coated with gold using a sputter coater [36,62].

### 3.5. In Vitro Cytotoxicity Studies

The cytotoxicity of PEI-TPP nanoparticles was evaluated on human embryonic kidney HEK293 cells as well as on human prostate PC3 and DU145 cells employing the standard MTT assay. Cells were cultured in RPMI 1640 medium supplemented with 10% FBS, L-glutamine (2 mM), and penicillin (100 U/mL)/streptomycin (100 μg/mL) solution, and incubated in a humidified atmosphere containing 5% CO_2_ at 37 °C. Cells were detached using a solution of trypsin (0.05% *w*/*v*) and EDTA (0.02% *w*/*v*), and then sub-cultured twice a week. The cells were inoculated in 96-well plates (10^4^ cells per well) and allowed to grow in a nutrient-rich solution containing 10% fetal bovine serum for a period of 24 h. Subsequently, cells were exposed to different doses of PEI-TPP for a duration of 3 h. Following incubation, the culture supernatants were disregarded and replaced with complete cell culture medium. After 24 h incubation time, the MTT assay was employed to evaluate the mitochondrial redox function, which is a measure of cell viability, in all cell groups. Concisely, the cell culture media were replaced with 100 μL of MTT solution (10 μg/mL in full medium) and then incubated at 37 °C in a 5% CO_2_ humidified environment for 4 h. Next, the MTT media were carefully removed, and the resulting formazan crystals were dissolved in 2-isopropanol (100 μL per well). An Infinite M200 microplate reader (Tecan group Ltd., Männedorf, Switzerland) was used to measure the absorbance at 540 nm. Blank values obtained from wells containing 2-isopropanol and no cells were subtracted in all instances. The relative cell viability was assessed as a percentage relative to untreated cells (control). Data were presented as the mean  ±  SD from at least three independent experiments in eight replicates for each concentration. Statistical analysis was performed using Student’s paired two-tailed *t*-tests to study the statistical significance between the treated cells and the control. Statistical significance follows the assignment * *p* < 0.05, ** *p* < 0.01, *** *p* < 0.001, **** *p* < 0.0001 and ns, not significant (*p* > 0.05).

## 4. Conclusions

In this study, hyperbranched polyethyleneimine (PEI) derivatives of two different molecular weights (1300 and 5000 Da) were functionalized with amphiphilic alkyl-triphenylphosphonium (alkylTPP) groups of two different alkyl chain lengths (C4, C10), known to induce membrane penetration, especially in cells with high membrane potential. The chemical structures of the PEI-TPP derivatives, namely PEI1300-TPP(C4), PEI1300-TPP(C10) PEI5000-TPP(C4) and PEI5000-TPP(C10), were characterized by FTIR and NMR spectroscopies, confirming the successful introduction of alkylTPP groups to the primary amino groups of PEI. Due to their lipophilic character, stable colloidal dispersions in various aqueous media were spontaneously obtained, which were subsequently physicochemically characterized by DLS and *ζ*-potential measurements. To investigate the antibacterial activity of PEI-TPP derivatives, Gram (−) *E. coli* and Gram (+) *S. aureus* bacteria were used. The effects of the polymer molecular weight, the alkyl chain length, and the number of triphenylphosphonium groups on their antibacterial efficacy were studied. The presence of alkylTPP groups significantly enhanced the antibacterial activities of parent PEI scaffolds leading to TPP-functionalized nanoparticles that exhibited concentration-dependent antibacterial activity against both tested bacteria strains, especially against *S. aureus*. Lower-molecular-weight polymeric derivatives and those with longer alkyl chains proved more efficient against both *E. coli* (MIC = 20 μg/mL) and *S. aureus* (MIC = 0.25 μg/mL). SEM images depicted changes in cell morphology, bacterial membrane disruption, and leakage of intracellular contents. These findings can be attributed to their lipophilicity and the known penetration ability of TPP-functionalized compounds through bacterial cell membranes, causing destabilization of membrane integrity, resulting in efficient internalization into bacterial cells and changing bacterial metabolic activity, and ultimately cell death. Moreover, cytocompatibility studies on human embryonic kidney HEK293 cells, as well as the human prostate PC3 and DU145 cells, revealed that polymeric derivatives bearing butylTPP groups were slightly more toxic (IC_50_ values of 60–100 μg/mL) than their decylTPP counterparts (IC_50_ ≥ 150 μg/mL). By comparing the antibacterial performance of all derivatives in combination with their cytotoxicity, PEI1300-TPP(C10) exhibited the best antibacterial performance (MIC = 20 μg/mL and 0.25 μg/mL for *E. coli* and *S. aureus*, respectively) as well as the lowest cytotoxicity at these relevant antibacterial concentrations (cell viability not statistically significant compared to the controls). Further studies concerning the safety of this derivative are needed. However, this study successfully highlights the importance of a structure–property relationship strategy in guiding the design of next-generation antibacterial polymers with enhanced efficacy and safety profiles.

## Figures and Tables

**Table 1:** Mean radii and *ζ*-potential value of PEI-TPP nanoparticles.

Samples	Mean Radius (nm)	*ζ*-Potential Value (mV)
PEI1300-TPP(C4)	80	+37_±2_
PEI5000-TPP(C4)	155	+58_±1_
PEI1300-TPP(C10)	40	+30_±2_
PEI5000-TPP(C10)	150	+60_±2_

**Table 2:** MIC and MBC values of PEI-TPPs against *E. coli* and *S. aureus* bacteria.

Samples	*E. coli*		*S. aureus*	
	MIC (μg/mL)	MBC (μg/mL)	MIC (μg/mL)	MBC (μg/mL)
PEI1300	150	400–500	100	300
PEI5000	300	500	100	300
PEI1300-TPP(C4)	150	400	50	300
PEI5000-TPP(C4)	100	300	25	150
PEI1300-TPP(C10)	20	25	0.25	5
PEI5000-TPP(C10)	40	50	0.25	5

## Data Availability

The data presented in this study are available on request from the corresponding author.

## Supplementary Materials

The supporting information can be downloaded at https://www.mdpi.com/article/10.3390/ijms26115153/s1.
