# Supplementary material for: Alkyltriphenylphosphonium-Functionalized Hyperbranched Polyethyleneimine Nanoparticles for Safe and Efficient Bacterial Eradication: A Structure–Property Relationship Study
Source: Int J Mol Sci. 2025 May 28;26(11):5153. doi: 10.3390/ijms26115153 (PMC12155293; doi:10.3390/ijms26115153)
Supplement: Supplementary file 1 [file ijms-26-05153-s001.zip › ijms-3568493-supplementary.pdf]

*Supplementary Information*

# Alkyltriphenylphosphonium-Functionalized Hyperbranched Polyethyleneimine Nanoparticles for Safe and Efficient Bacterial Eradication: A Structure–Property Relationship Study

**Katerina N. Panagiotaki, Kyriaki-Marina Lyra, Aggeliki Papavasiliou, Dimitris Tsiourvas and Zili Sideratou ***

Institute of Nanoscience and Nanotechnology, National Centre of Scientific Research "Demokritos", 15310 Aghia Paraskevi, Attiki, Greece; k.panagiotaki@inn.demokritos.gr (K.N.P.); k.lyra@inn.demokritos.gr (K.-M.L.); a.papavasiliou@inn.demokritos.gr (A.P.); d.tsiourvas@inn.demokritos.gr (D.T.)

* Correspondence: z.sideratou@inn.demokritos.gr; Tel.: +30-210-6503616

**Table of Contents**

**Figure S1**: $^{1}H$ and $^{13}C$ NMR spectra of PEI1300-TPP(C4).

**Figure S2**: $^{1}H$ and $^{13}C$ NMR spectra of PEI5000-TPP(C4).

**Figure S3**: $^{1}H$ and $^{13}C$ NMR spectra of PEI1300-TPP(C10).

**Figure S4**: $^{1}H$ and $^{13}C$ NMR spectra of PEI5000-TPP(C10).

**Figure S5**: FTIR spectra of PEI-TPP derivatives.

**Figure S6.** Intensity weighted hydrodynamic radii size distributions of PEI1300-TPP(C4) (**A**), PEI1300-TPP(C10) (**B**), PEI5000-TPP(C4) (**C**), and PEI5000-TPP(C10) (**D**) nanoparticles.

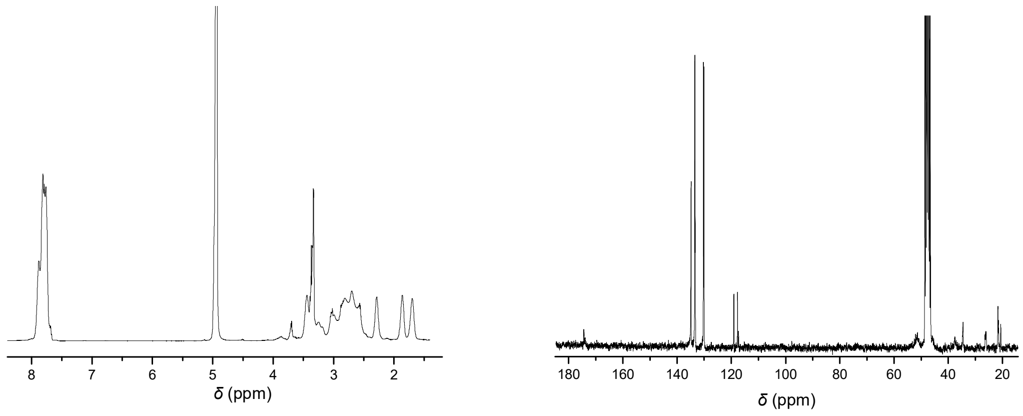


**Figure S1**: $^{1}$H and $^{13}$C NMR spectra of PEI1300-TPP(C4).

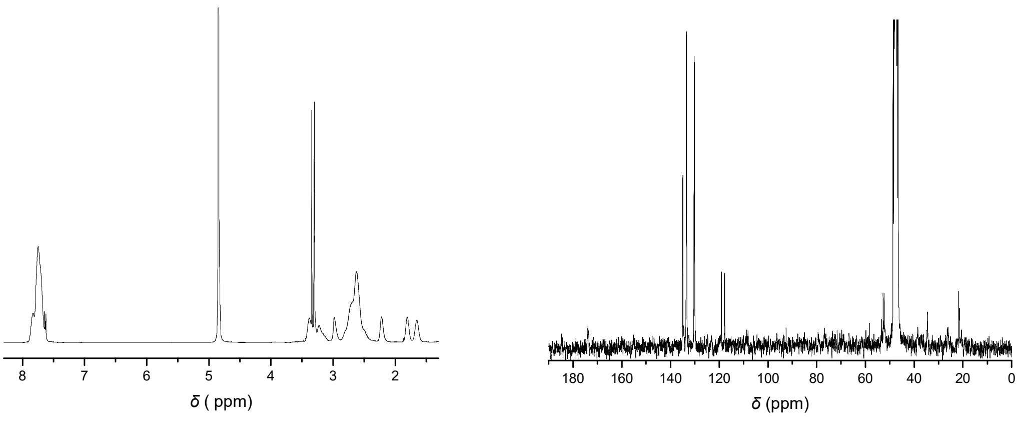


**Figure S2**: $^{1}$H and $^{13}$C NMR spectra of PEI5000-TPP(C4).

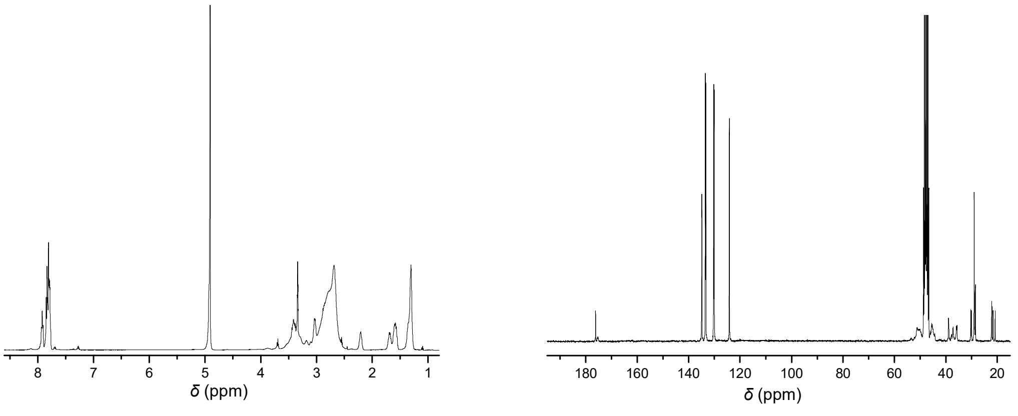


**Figure S3**: $^{1}$H and $^{13}$C NMR spectra of PEI1300-TPP(C10).

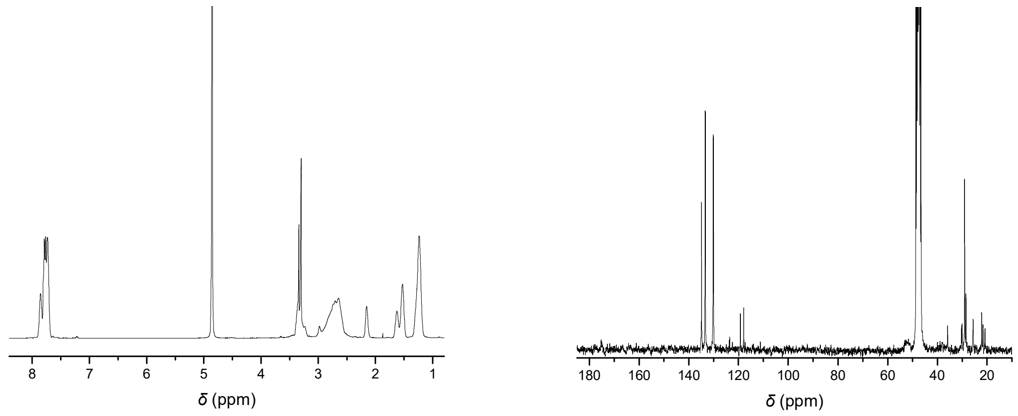


**Figure S4**: $^{1}H$ and $^{13}C$ NMR spectra of PEI5000-TPP(C10).

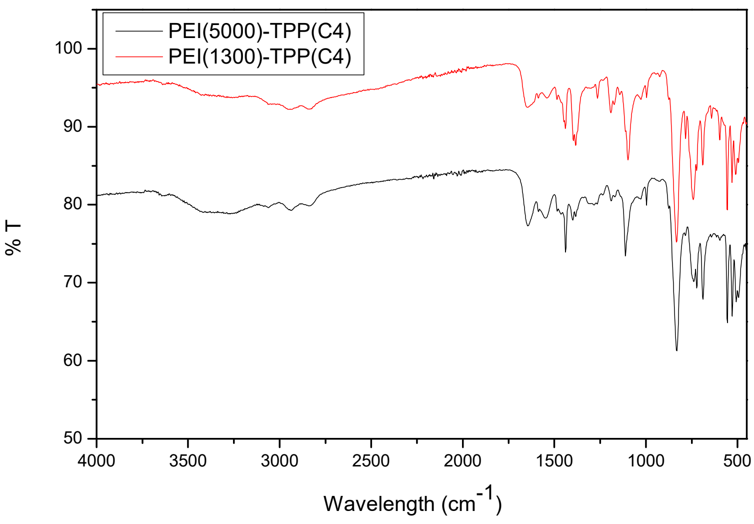


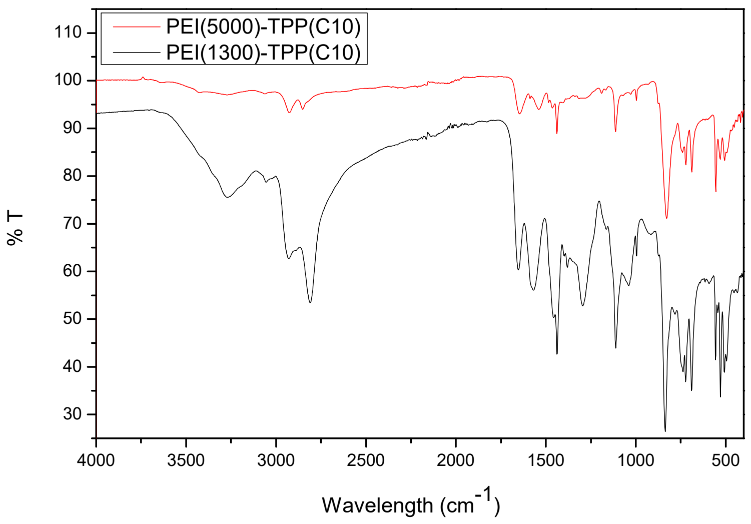


**Figure S5**: FTIR spectra of PEI-TPP derivatives.

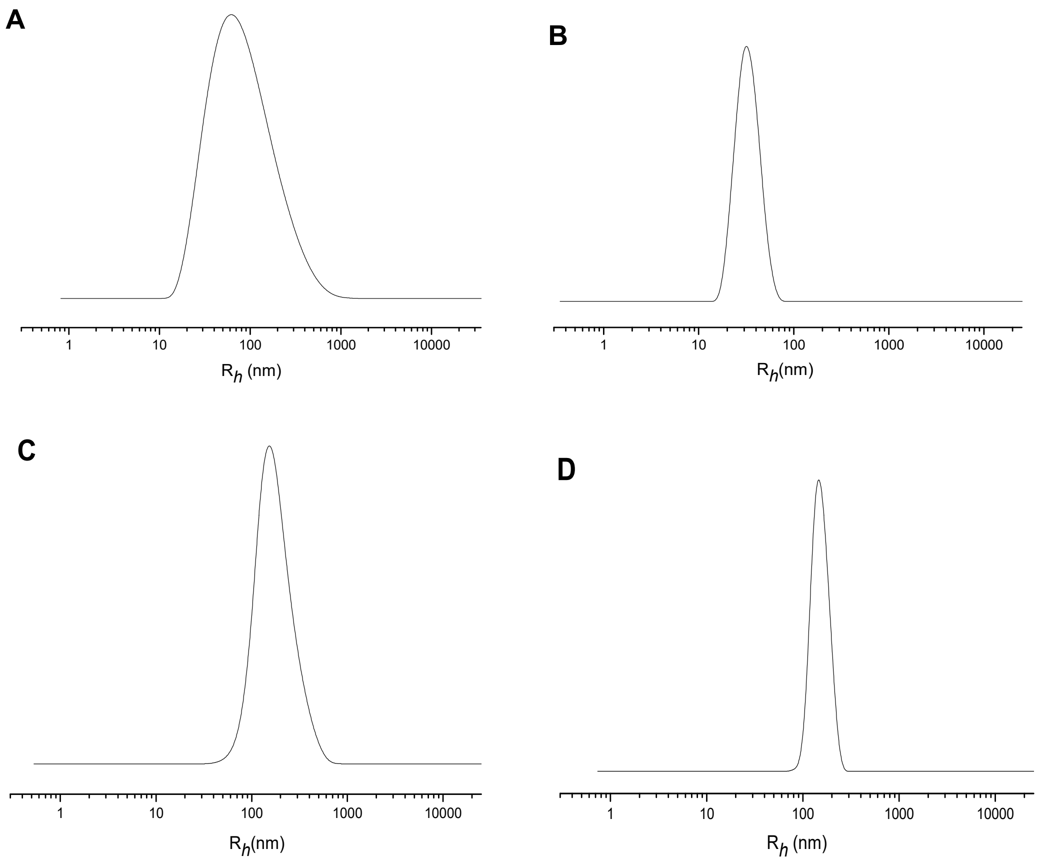


**Figure S6.** Intensity weighted hydrodynamic radii size distributions of PEI1300-TPP(C4) (**A**), PEI1300-TPP(C10) (**B**), PEI5000-TPP(C4) (**C**), and PEI5000-TPP(C10) (**D**) nanoparticles.
